# Highlight: The Surprising Endurance of DNA's Smallest Repeats

**DOI:** 10.1093/gbe/evae044

**Published:** 2024-03-28

**Authors:** Casey McGrath

Microsatellites are valuable tools for studying inheritance, genetic diversity, and population dynamics across a wide range of organisms including bacteria, plants, animals, and fungi. These short, repeating sequence motifs are a common feature of both coding and noncoding DNA and have been observed in all genomes studied to date. Their repetitive nature leads to “slippage” in the DNA replication machinery, resulting in the addition or subtraction of repeats that causes microsatellites to grow or shrink in length. Because of this, there is considerable variability among individuals in the number of repeats at each microsatellite locus. A well-known microsatellite locus is the string of “CAG” nucleotides in the human huntingtin gene, which leads to the development of Huntington's disease in individuals with more than 37 copies of the repeat. Despite being widely used in population and evolutionary biology, the evolutionary fates of microsatellites themselves remain hotly debated. In a new study in *Genome Biology and Evolution*, titled “Ancient and modern genomes reveal microsatellites maintain a dynamic equilibrium through deep time,” an international team of researchers used a unique data set of modern and ancient Adélie penguin genomes to uncover new insights into the evolution of microsatellites (McComish et al. 2024). Led by David Lambert from Griffith University, the study reveals the remarkable persistence and stability of microsatellites over vast evolutionary time.

To study long-term microsatellite dynamics, the study's authors sequenced the genomes of 23 ancient Adélie penguin specimens dating back over 46,000 yr, as well as samples from 26 modern Adélie penguins (Fig. 1), enabling a direct comparison between ancient and modern individuals, a situation that remains relatively uncommon among evolutionary studies. “Ancient DNA provides a unique opportunity to look at old problems in new ways,” note the study's authors. “In contrast to the traditional method of comparing living representatives of different taxa, ancient DNA gives us the opportunity to ‘step back in time’.” The researchers further compared this data set with over 27 million microsatellite loci from 63 other animal genomes, providing a look at microsatellite dynamics over more than 500 million years.

**Fig. 1.**
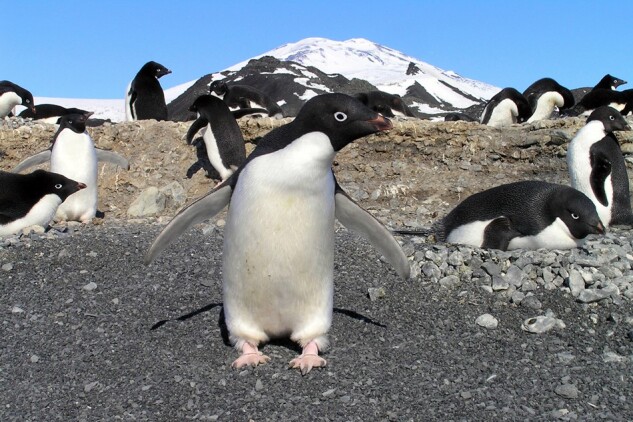
Photograph of an Adélie penguin breeding colony, Edmonson Point, Antarctica. Adélie penguin nests consist of a shallow scrape, pebbles, and bones. Credit: Carlo Baroni and Maria Cristina Salvatore, University of Pisa.

One of the most surprising findings of the study was that microsatellite length remained extremely stable over thousands and even millions of years. This answers a long-held question about whether microsatellites tend to get longer or shorter over time. “We were all surprised at the lack of evidence for an upward genetic drift in microsatellite repeat length,” says the research team, comprising authors from Australia, the United States, Italy, China, Denmark, and New Zealand. “At the outset of this project, it seemed likely that microsatellite alleles would have arisen as ‘pure’ short repeats [i.e., perfect repeats with no errors in the repeat pattern]. Over time, we expected that these alleles would increase in length until enough point mutations effectively disrupted the repeat structure and stopped the upward drift. This cycle could be characterized as a general pattern of birth, growth, decay, and finally the death of the microsatellite.”

Contrary to this expectation, however, the researchers found that microsatellites tend to grow by an average of only one nucleotide every 100 million years. This remarkable stability over time suggests a dynamic equilibrium in the replication slippage process that generates length polymorphism; in other words, longer microsatellites tend to get shorter, and shorter microsatellites tend to get longer, maintaining microsatellite length over time.

The authors were also surprised by how extraordinarily long-lived some microsatellites are: “We found that some microsatellite loci persisted for over half a billion years and therefore had survived many speciation events.” The persistence and stability of microsatellites is especially notable given the high variability of microsatellite length observed among individuals. According to the authors, one possibility is that “microsatellites might play a functional role in the architecture of the genome or in generating phenotypic diversity, as it seems unlikely that they would persist for so long unless they were being protected from degeneration by purifying selection.”

The genomes of the ancient and modern Adélie penguins sequenced in the study will provide an incredible resource for future research, enabling investigation into more complex models of microsatellite evolution that include both point mutations and slippage. These data will likely be useful for investigating the evolution of other types of genetic elements and repetitive DNA sequences. The creation of this rich data set would not have been possible without the efforts of coauthors Carlo Baroni and Maria Cristina Salvatore, who spent decades recovering the subfossil remains of Adélie penguins from the Ross Sea area of Antarctica to characterize the changing climatic conditions of the region. “Their research has been very important to us,” note their coauthors, “and it has only been thanks to what might appear to be, on the face of it, an unlikely collaboration that we have been able to develop this exciting research project.”

## References

[evae044-B1] McComish BJ , Charleston MA, Parks M, Baroni C, Salvatore MC, Li R, Zhang G, Millar CD, Holland BR, Lambert D. Ancient and modern genomes reveal microsatellites maintain a dynamic equilibrium through deep time. Genome Biol Evol. 2024:16:evae017. 10.1093/gbe/evae017. 38412309 PMC10972684

